# Supplementary material for: Quinoxaline-based anti-schistosomal compounds have potent anti-plasmodial activity
Source: PLoS Pathog. 2025 Feb 3;21(2):e1012216. doi: 10.1371/journal.ppat.1012216 (PMC11809919; doi:10.1371/journal.ppat.1012216)
Supplement: S1 Fig — (PDF) [file ppat.1012216.s001.pdf]

Compound 22

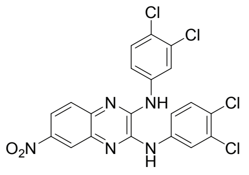

Compound 22c

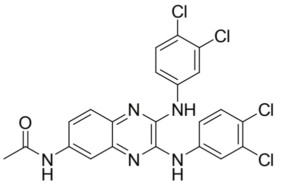

Compound 22f

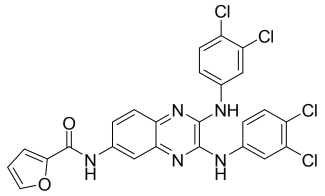

Compound 25

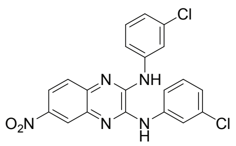

Compound 26

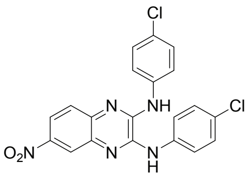

Compound 30

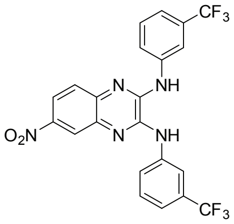

Compound 31

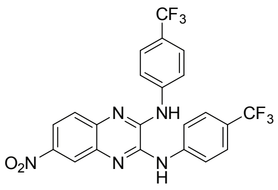

Compound 32

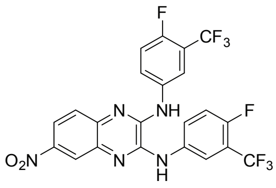

Compound 33

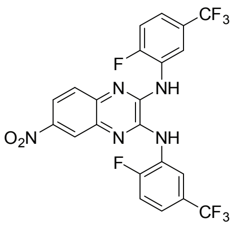

Compound 35

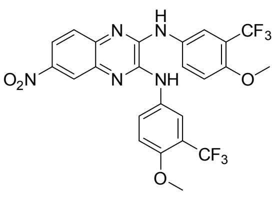

Compound 37

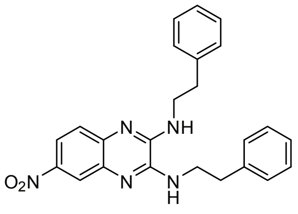

S1 Fig: Structures of compounds
