# Supplementary material for: Quinoxaline-based anti-schistosomal compounds have potent anti-plasmodial activity
Source: PLoS Pathog. 2025 Feb 3;21(2):e1012216. doi: 10.1371/journal.ppat.1012216 (PMC11809919; doi:10.1371/journal.ppat.1012216)
Supplement: S2 Fig — Stage-specific dose response assays for compounds (A) 22, (B) 31, and (C) 33. Assays were performed on tightly synchronised cultures, with compound applied for 10 h at the ring (6–16 h post-invasion), trophozoite (18–28 h post-invasion) and schizont (30–40 h post-invasion) stages. After drug washout, cultures were incubated for a further 62 h. For comparison, assays were also performed with constant drug pressure applied for the full 72 h. The bar graph (left) represents mean IC50 values for three replicates, the survival graphs (right) display the dose response curves from three independent experiments. Error bars indicate the standard error of the mean based on these three independent repeats. (PDF) [file ppat.1012216.s002.pdf]

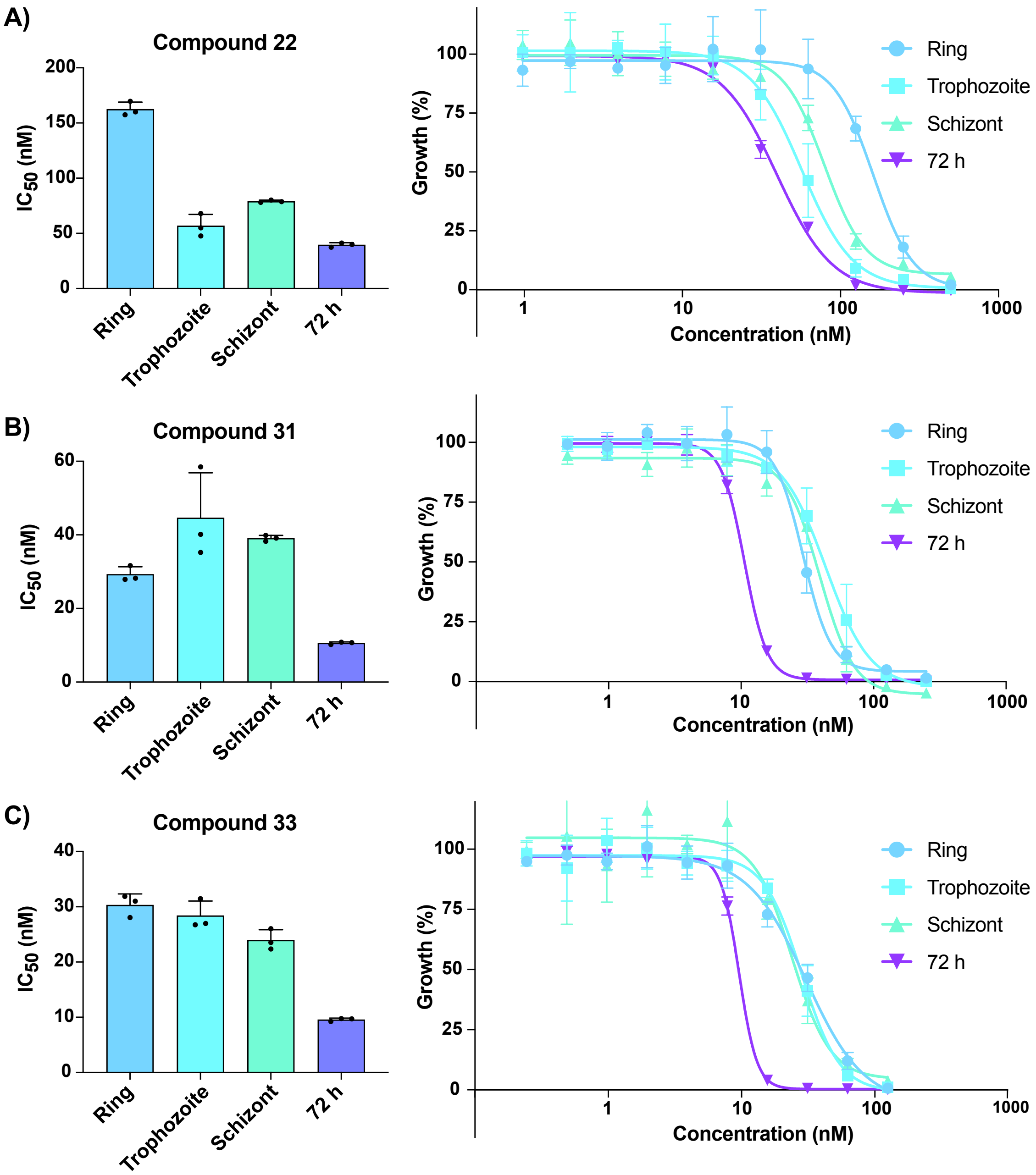

S2 Fig: Stage-specificity profiling

Stage-specific dose response assays for compounds (A) **22**, (B) **31**, and (C) **33**. Assays were performed on tightly synchronised cultures, with compound applied for 10 h at the ring (6-16 h post-invasion), trophozoite (18-28 h post-invasion) and schizont (30-40 h post-invasion) stages. After drug washout, cultures were incubated for a further 62 h. For comparison, assays were also performed with constant drug pressure applied for the full 72 h. The bar graph (*left*) represents mean IC_{50} values for three replicates, the survival graphs (*right*) display the dose response curves from three independent experiments. Error bars indicate the standard error of the mean based on these three independent repeats.
