# Supplementary material for: Quinoxaline-based anti-schistosomal compounds have potent anti-plasmodial activity
Source: PLoS Pathog. 2025 Feb 3;21(2):e1012216. doi: 10.1371/journal.ppat.1012216 (PMC11809919; doi:10.1371/journal.ppat.1012216)
Supplement: S3 Fig — Rate of parasite killing for compounds 22, 31 and 33 was examined by treating Dd2 parasites in unlabelled RBCs with 10×IC50 for 24 and 48 h, followed by washout. Reinvasion of viable parasites into fresh RBCs pre-labelled with carboxylfluorescein diacetate succinimidyl ester (CFDA-SE) was measured after a further 48 h, and the percentage of remaining viable parasites compared with untreated controls. For comparison, standard antimalarials included fast-killing dihydroartemisinin, chloroquine and slow-killing atovaquone. The average (±SD) of three biological replicates (each with two technical replicates) is shown. (PDF) [file ppat.1012216.s003.pdf]

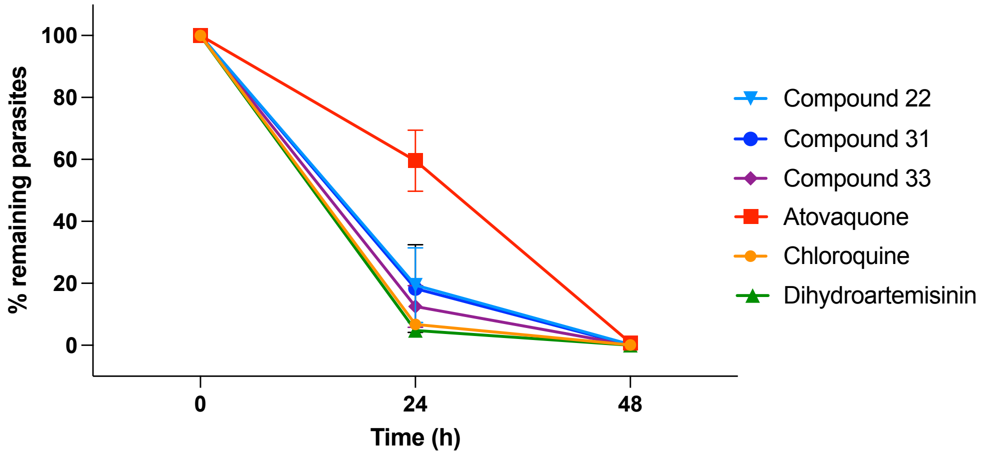

S3 Fig: Parasite killing rate

Rate of parasite killing for compounds **22**, **31** and **33** was examined by treating Dd2 parasites in unlabelled RBCs with 10xIC_{50} for 24 and 48 h, followed by washout. Reinvasion of viable parasites into fresh RBCs pre-labelled with carboxylfluorescein diacetate succinimidyl ester (CFDA-SE) was measured after a further 48 h, and the percentage of remaining viable parasites compared with untreated controls. For comparison, standard antimalarials included fast-killing dihydroartemisinin, chloroquine and slow-killing atovaquone. The average (±SD) of three biological replicates (each with two technical replicates) is shown.
