# Supplementary material for: Quinoxaline-based anti-schistosomal compounds have potent anti-plasmodial activity
Source: PLoS Pathog. 2025 Feb 3;21(2):e1012216. doi: 10.1371/journal.ppat.1012216 (PMC11809919; doi:10.1371/journal.ppat.1012216)
Supplement: S4 Fig — (A) Comparison of compound potency between (left) the larval stage of S. mansoni and P. falciparum 3D7 strain, and (right) HepG2 and P. falciparum 3D7. Data for S. mansoni and HepG2 were derived from [13] and are shown in S2 Table. Correlation was performed using Spearman’s correlation test. (B) Selectivity index of compounds on P. falciparum 3D7 or Dd2 strains was calculated relative to HepG2 72 h CC50 values (S2 Table). (PDF) [file ppat.1012216.s004.pdf]

A)

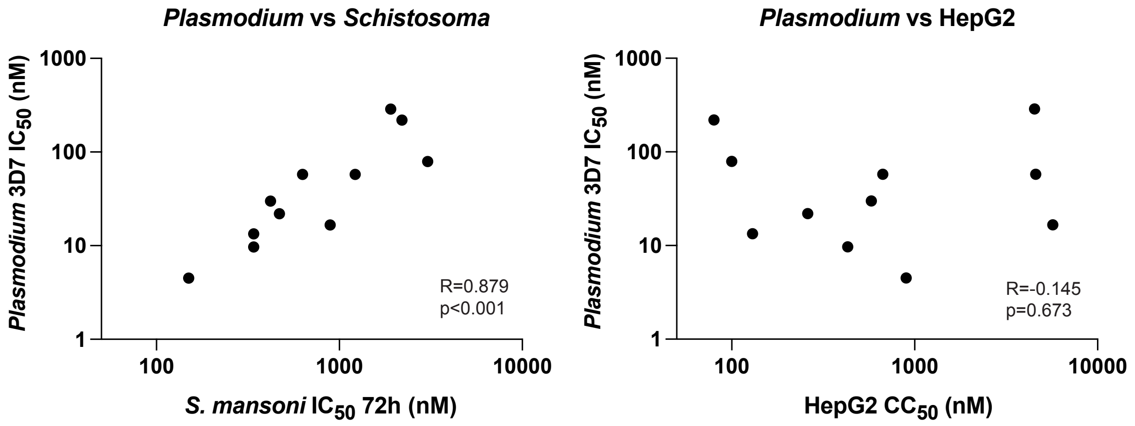

B)

Selectivity index (HepG2 CC_{50}/ Pf IC_{50})		
**Compound**	**HepG2/3D7**	**HepG2/Dd2**
22	11.9	8.0
22c	58.1	25.4
22f	19.8	14.9
25	10.1	9.2
26	4.3	3.5
30	67.2	40.9
31	8.2	5.7
32	40.4	24.1
33	22.2	14.1
35	7.4	3.8
37	20.5	17.1

S4 Fig: Selectivity of compounds

A) Comparison of compound potency between (*left*) the larval stage of *S. mansoni* and *P. falciparum* 3D7 strain, and (*right*) HepG2 and *P. falciparum* 3D7. Data for *S. mansoni* and HepG2 derived from [13] and shown in **S2 Table**. Correlation was performed using Spearman's correlation test. **B)** Selectivity index of compounds on *P. falciparum* 3D7 or Dd2 strains was calculated relative to HepG2 72 h CC_{50} values (**S2 Table**).
