# Supplementary material for: Quinoxaline-based anti-schistosomal compounds have potent anti-plasmodial activity
Source: PLoS Pathog. 2025 Feb 3;21(2):e1012216. doi: 10.1371/journal.ppat.1012216 (PMC11809919; doi:10.1371/journal.ppat.1012216)
Supplement: S5 Fig — Clones derived from in vitro resistance evolution with compound 22 (see Fig 2) were evaluated against additional compounds from this series, compounds 30, 32, 33 and 35. Compound 30 is a structural isomer of compound 31 (Fig 2b) and compounds 32 and 33 are isomers of each other. Each dot represents a biological replicate (n = 5) with mean±SD shown as bar chart, and statistical significance determined by Mann-Whitney U test (*p < 0.05, **p < 0.01). (PDF) [file ppat.1012216.s005.pdf]

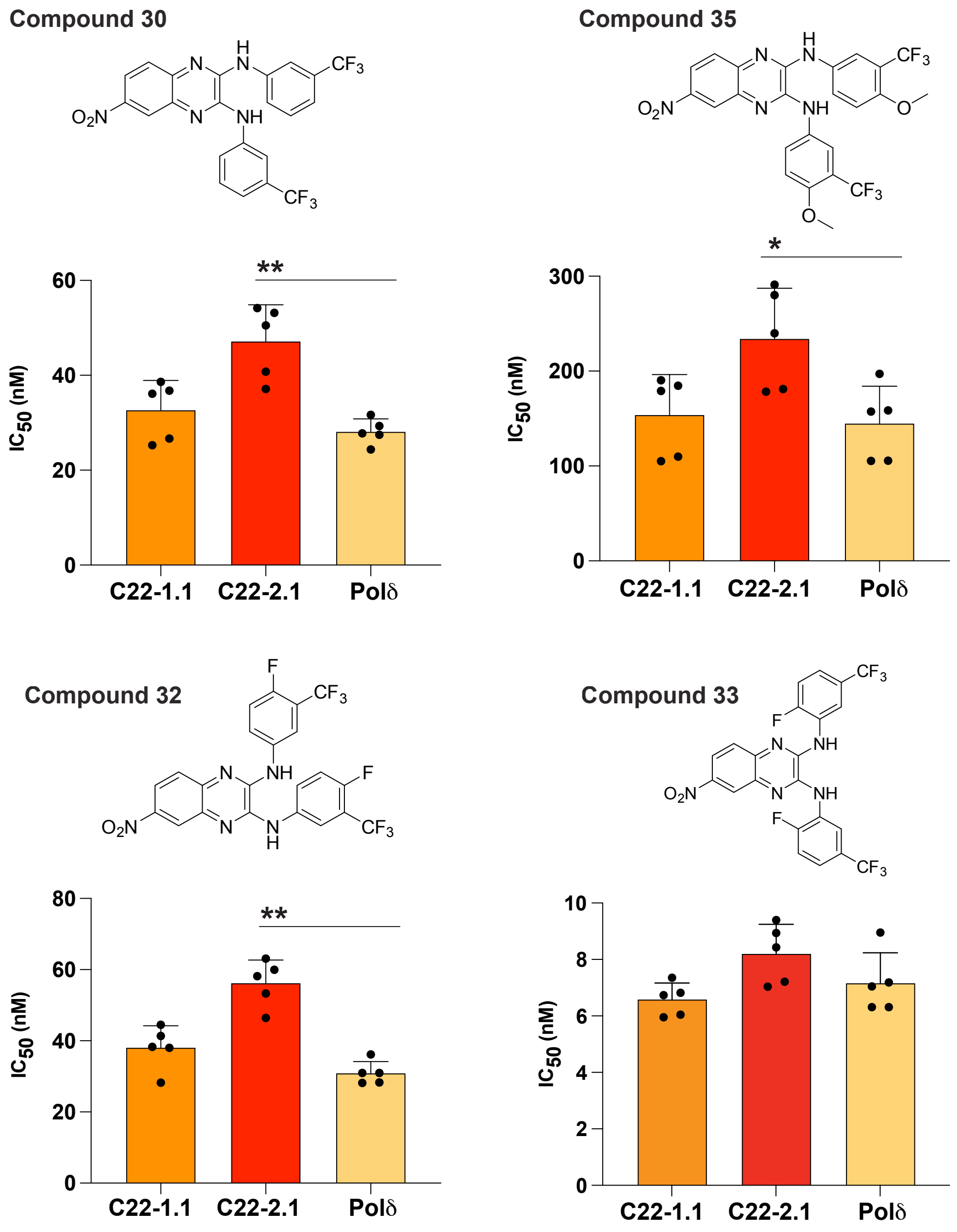

S5 Fig: Cross-resistance of compound 22-evolved clones

Clones derived from *in vitro* resistance evolution with compound **22** (see **Fig. 2**) were evaluated against additional compounds from this series, compounds **30**, **32**, **33** and **35**. Compound **30** is a structural isomer of compound **31** (Fig.2b) and compounds **32** and **33** are isomers of each other. Each dot represents a biological replicate (n=5) with mean±SD shown as bar chart, and statistical significance determined by Mann-Whitney *U* test (*p<0.05, **p<0.01).
