# Supplementary material for: Quinoxaline-based anti-schistosomal compounds have potent anti-plasmodial activity
Source: PLoS Pathog. 2025 Feb 3;21(2):e1012216. doi: 10.1371/journal.ppat.1012216 (PMC11809919; doi:10.1371/journal.ppat.1012216)
Supplement: S6 Fig — Three independent cultures of either the parental Dd2 or isogenic Dd2-Polδ mutator lines were exposed to compound 22 or GNF179. Parasite inoculum ranged from 104 to 108 parasites per culture. Drug pressure was initiated at 3×IC50 for both compound 22 (90 nM) and GNF179 (10.5 nM). For compound 22, the selection pressure was raised to 4×IC50 (120 nM) at day 6 and 5×IC50 (150 nM) at day 9 in order to fully clear the culture. Red squares indicate absence of parasites after 45 days (GNF179) or 54 days (compound 22), green squares indicate recrudescence, with the day of recrudescence representing the day when parasitemia crossed 1%. (PDF) [file ppat.1012216.s006.pdf]

A)

Compound 22						
Dd2 wildtype	**Parasite inoculum**	**10^4**	**10^5**	**10^6**	**10^7**	**10^8**
	Replicate 1					
	Replicate 2					
	Replicate 3					
	Recrudescent day					
Dd2-Polδ	**Parasite inoculum**	**10^4**	**10^5**	**10^6**	**10^7**	**10^8**
	Replicate 1					
	Replicate 2					
	Replicate 3					
	Recrudescent day					

B)

GNF179						
Dd2 wildtype	**Parasite inoculum**	**10^4**	**10^5**	**10^6**	**10^7**	**10^8**
	Replicate 1					
	Replicate 2					
	Replicate 3					
	Recrudescent day				**26-42**	**26-32**
Dd2-Polδ	**Parasite inoculum**	**10^4**	**10^5**	**10^6**	**10^7**	**10^8**
	Replicate 1					
	Replicate 2					
	Replicate 3					
	Recrudescent day			**35**	**16**	**26**

S6 Fig: Minimum inoculum for resistance (MIR).

Three independent cultures of either the parental Dd2 or isogenic Dd2-Polδ mutator lines were exposed to compound **22** or GNF179. Parasite inoculum ranged from 10^4 to 10^8 parasites per culture. Drug pressure was initiated at 3×IC_{50} for both compound **22** (90 nM) and GNF179 (10.5 nM). For compound **22**, the selection pressure was raised to 4×IC_{50} (120 nM) at day 6 and 5×IC_{50} (150 nM) at day 9 in order to fully clear the culture. Red squares indicate absence of parasites after 45 days (GNF179) or 54 days (compound **22**), green squares indicate recrudescence, with the day of recrudescence representing the day when parasitemia crossed 1%.
