# Supplementary material for: Quinoxaline-based anti-schistosomal compounds have potent anti-plasmodial activity
Source: PLoS Pathog. 2025 Feb 3;21(2):e1012216. doi: 10.1371/journal.ppat.1012216 (PMC11809919; doi:10.1371/journal.ppat.1012216)
Supplement: S7 Fig — Representative plot showing inhibition of β-hematin formation versus concentration profiles for MMV007224 (black), MMV665794 (red), chloroquine (blue) and KAE609 (green). Adjacent IC50 data represent mean values of two biological replicates performed with technical duplicates. Data were fitted to the sigmoidal concentration response (variable slope) equation in GraphPad Prism to determine the IC50. (PDF) [file ppat.1012216.s007.pdf]

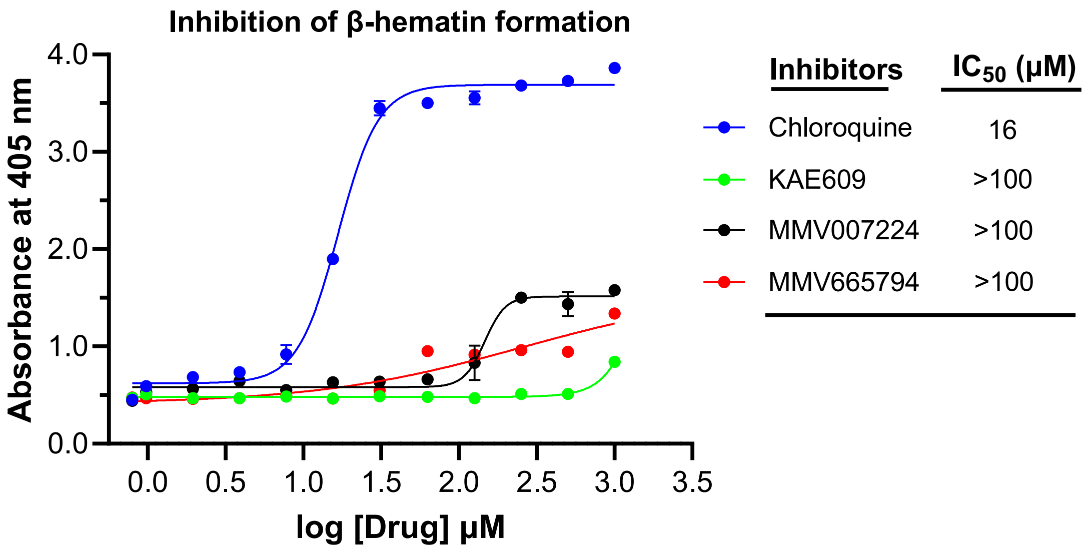


**S7 Fig: β-hematin inhibition assay for quinoxaline analogs and antimalarial control compounds**. Representative plot showing inhibition of β-hematin formation versus concentration profiles for MMV007224 (black), MMV665794 (red), chloroquine (blue) and KAE609 (green). Adjacent $IC_{50}$ data represents mean values of two biological replicates performed with technical duplicates. Data were fitted to the sigmoidal concentration response (variable slope) equation in GraphPad Prism to determine the $IC_{50}$.
