# Supplementary material for: Quinoxaline-based anti-schistosomal compounds have potent anti-plasmodial activity
Source: PLoS Pathog. 2025 Feb 3;21(2):e1012216. doi: 10.1371/journal.ppat.1012216 (PMC11809919; doi:10.1371/journal.ppat.1012216)
Supplement: S8 Fig — (A) Lipid flippases used in the alignment. (B) Sequence alignment of human ATP8A2, P. falciparum PfATP2, and six putative lipid-translocating ATPases from S. mansoni. The actuator (A), nucleotide binding (N), and phosphorylation (P) domains are shown, as well as the first six transmembrane segments (M1-6). Key conserved residues D (in A domain) and D (P domain) involved in the phosphorylation (DKTGT) and dephosphorylation (DGET) cycle are highlighted by a star. The purple circles highlight the conserved N and I residues located in M4 domain that are important for recognition and release of lipid, respectively. The green triangle indicates the K residues in the M5 domain required for the sensitivity to the lipid subtype [26,47]. (PDF) [file ppat.1012216.s008.pdf]

A

Organism	Gene ID	Gene name	Accession no.
Human	ATP8A2	ATP8A2	Q9NTI2
P. falciparum	PF3D7_1219600	ATP2	Q8I5L4
S. mansoni	Smp_091650	Smp_091650	A0A3Q0KJ05
S. mansoni	Smp_104500	Smp_104500	A0A5K4EK18
S. mansoni	Smp_163820	Smp_163820	A0A5K4EW97
S. mansoni	Smp_181230	Smp_181230	A0A3Q0KTZ9
S. mansoni	Smp_332390	Smp_332390	A0A5K4F868
S. mansoni	Smp_333250	Smp_333250	A0A5K4FA05

B

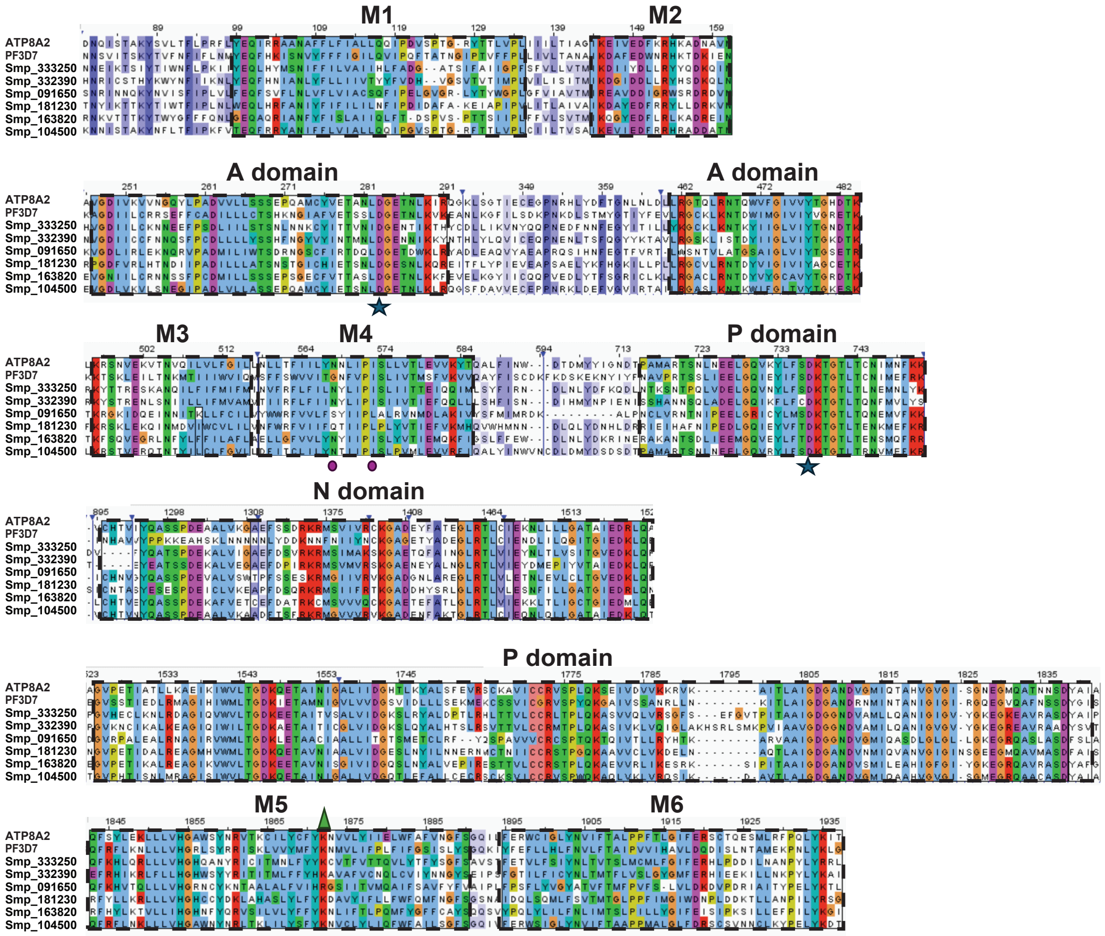

Supplementary Figure 8: Sequence alignment of phospholipid flippases. **A)** Lipid flippases used in the alignment. **B)** Sequence alignment of human ATP8A2, *P. falciparum* PfATP2, and six putative lipid-translocating ATPases from *S. mansoni*. The actuator (A), nucleotide binding (N), and phosphorylation (P) domains are shown, as well as the first six transmembrane segments (M1-6). Key conserved residues D (in A domain) and D (P domain) involved in the phosphorylation (DKTGT) and dephosphorylation (DGET) cycle are highlighted by a star. The purple circles highlight the conserved N and I residues located in M4 domain that are important for recognition and release of lipid, respectively. The green triangle indicates the K residues in the M5 domain required for the sensitivity to the lipid subtype [26, 45].
