# Supplementary material for: Quinoxaline-based anti-schistosomal compounds have potent anti-plasmodial activity
Source: PLoS Pathog. 2025 Feb 3;21(2):e1012216. doi: 10.1371/journal.ppat.1012216 (PMC11809919; doi:10.1371/journal.ppat.1012216)
Supplement: Attachment — Submitted filename: Reviewer comments_Response_final.pdf [file ppat.1012216.s016.pdf]

Response to reviewer questions

We would like to thank all reviewers for their constructive input. For this revision, we have included a substantial amount of new data in the manuscript, including evaluating the compounds against additional parasite strains (**Fig.1c**), testing stage-specificity during the asexual blood stage (**Supp.Fig.2**), measuring rate of kill (**Supp.Fig.3**), showing toxicity/selectivity index of the compounds (**Supp.Fig.4**), performing a minimum inoculation for resistance study (**Supp.Fig.6**), testing the impact on hemoglobin degradation (**Fig.6** and **Supp.Fig.7**) and evaluating compound 33 and additional related analogs against the QRP1 mutant and other resistant lines (**Supp.Fig.5**, **Fig.4c**, **Fig.5c**). In addition, we have added supplementary tables showing Lipinski rule of five criteria and predicted ADME properties for the compounds (**Supp. Tables 3** and **4**), and have made numerous textual changes in response to the reviewer comments and to improve clarity.

Note that references to page / paragraph numbers refers to the tracked-changes version of the manuscript.

Reviewer #1:

Part I - Summary

Please use this section to discuss strengths/weaknesses of study, novelty/significance, general execution and scholarship.

Reviewer #1: This report addresses antimalarial properties of quinoxalines originally developed to treat schistosomiasis. The MS is well-written, clearly describes potent compounds with limited ability to select for parasite resistance, and offers a partial explanation of the mechanism of resistance to one compound. The conclusions build on prior work by the authors in which they studied selection of resistance by related compounds. Some concerns are as follows.

Part II – Major Issues: Key Experiments Required for Acceptance

Please use this section to detail the key new experiments or modifications of existing experiments that should be absolutely required to validate study conclusions.

Reviewer #1: 1) The antimalarial properties of the compounds studied were confined to simple in vitro IC50 determinations. In depth consideration of antimalarial or drug-like properties of the compounds were not considered. An antimalarial drug discovery report would typically contain a good deal more data on activity, e.g. activity against additional parasite strains, stage specificity of activity, rate of killing, activity against other parasite stages, and/or in vivo activity in a murine model. Enthusiasm for this compound series (and thus interest in studies of mechanisms of resistance) would be greater if the promise of the class as potential antimalarials was better established.

We have now added additional data related to this compound series.

i) Using compounds **22**, **31**, and **33**, we have performed additional dose response assays against a more geographically diverse set of parasite strains. Supplementing existing data with the standard lab lines 3D7 and Dd2 as well as two Cambodian isolates, we now include data on strains from Uganda, Tanzania and Ghana. These data (Figure 1c) show the compounds appear equally potent across all strains tested with no significant differences between strains.

ii) We have performed stage-specificity assays with compounds **22**, **31** and **33**, evaluating them against ring, trophozoite and schizont stage parasites using pulse/washout assays essentially as described in (Murithi et al., 2020, Cell Chem Biol). These data are shown in new Supp. Fig. 2.

iii) We have evaluated the parasite rate of kill, compared with standard fast- and slow-killing antimalarials (new Supp. Fig. 3).

iv) We examined the potential of the quinoxaline-analogs to perturb the hemoglobin degradation pathway (new Fig.6 and Supplementary Fig.7).

v) We have cited previous data on the related analog MMV007224 indicating a lack of activity against liver-stage parasites (Meister et al., 2011, Science), alongside a wider discussion about previous studies on *atp2* essentiality and the inability to determine whether *atp2* is essential at the liver stage (Discussion, page 12 paragraph 1 in the tracked-changes document).

2) Similarities between inhibition of malaria parasites and schistosome worms is interesting, but this begs questions about the specificity of the inhibitors. Malaria parasites are arguably as different from schistosomes as both organisms are from humans. The manuscript offers no information, either from described new experiments or the literature, on the likelihood that the studied quinoxalines will exert toxicity in humans, as they do in protozoans and worms. Indeed, this omission is glaring and almost seems intentional; one suspects that there are skeletons in the closet. Some description of data suggesting a therapeutic window, such that quinoxalines can be seriously considered as drugs, would be very helpful. No toxicity data or other considerations of drug-like (ADME) properties of the compounds is included in this report, but if some such data was included in prior studies of schistosomes (or any other literature), reference to that literature would be helpful.

Apologies for not highlighting the HepG2 cytotoxicity data – this was included in the original submission in Supp. Table 1 alongside the *Plasmodium* and *Schistosoma* data for comparative purposes, but as the HepG2 data was originally published in the Padalino et al. study (2021, *Eur J.Med.Chem*.) we did not sufficiently highlight it in this current manuscript. We have now added a reference to this in the Results (page 5, paragraph 3), and have generated a new Supplementary Figure (Supp. Fig. 4) showing activity correlation between *P. falciparum* and HepG2, as well as selectivity index. In comparison with 3D7, the selectivity index ranges from 4.3 to 67, which is encouraging for a hit compound and suggests there are opportunities to further improve the range. We have also included consideration of Lipinski rule of five criteria and predicted ADME properties, inserting a description of this into the Results (page 5 paragraph 3) and adding Supplemental Tables 3 and 4.

3) Consideration of ease of resistance selection and insights into resistance mechanisms form the bulk of the manuscript. It is not clear why the authors did not study selection of resistance in standard *P. falciparum* strains, but rather only in an engineered strain. This strain is a great tool to study compounds for which resistance cannot easily be selected (as appears to be the case with the quinoxalines), but it would have been preferable to first test ease of selection of resistance using a standard approach, with a somewhat lower concentration of compound tested against varied

numbers of parasites; excellent, now standardized approaches for such analyses have been developed by one of the authors (David Fidock). Although the results certainly suggest that the tested compounds do not easily select for resistance, it would have been helpful to have direct comparison of ease of selection using a standard approach, which would have allowed comparison with a large literature describing similar experiments with dozens of other compounds, and also to include controls (e.g. atovaquone, which selects easily) in the described experiments. Nonetheless, the selection experiments were of value, as successful selection of resistance with compound 22 enabled studies of mechanisms of resistance selection.

We have now included minimum inoculum for resistance (MIR) assays performed with both wildtype Dd2 strain and the isogenic Dd2-Polδ mutator line for direct comparison. Using a range of parasite inocula ranging from 10^4 to 10^8, in triplicate cultures, resistance selections with compound **22** resulted in the absence of recrudescent parasites across all inocula. In contrast, a control compound GNF179 (related to the clinical candidate KAF156/ganaplacide) yielded resistance at 10^7 for the wildtype Dd2 line and 10^6 for the isogenic Dd2-Polδ mutator line (see new Supp. Fig. S6, as well as Results page 7, paragraph 2).

Regarding selection concentration, we initially attempted to perform the MIR selections with compound **22** at 3xIC_{50}, however after six days at 3×IC_{50} and a further 3 days at 4×IC_{50}, the culture had failed to fully clear and thus it was necessary to increase the concentration to 5×IC_{50}. Nonetheless, even this higher concentration (5×30nM = 150nM) is lower than the current standard MIR selection protocol used by MMV of 3×IC_{90} (Duffey et al., 2021, Trends Parasit., PMID: 34001441), which would equate to approximately 250nM for compound **22**.

Part III – Minor Issues: Editorial and Data Presentation Modifications
Please use this section for editorial suggestions as well as relatively minor modifications of existing data that would enhance clarity.

Reviewer #1: 1) Line 26. "Evolution of resistance" is shorthand that will not be understood by many readers. Wording should be changed to explain that experiments involved attempts to select for resistance in vitro.

This was modified to "In vitro resistance selections" in the Abstract. We have further detailed this approach in the Introduction (see below).

2) Line 43. "New combinations of drugs" is not a strategy. The strategy is to utilize new drugs for the treatment or prevention of malaria.

This phrase was removed and the sentence rewritten.

3) Line 45. The development of effective drugs does not require identification of targets. Many drugs that effectively treat malaria and other infections do not have known targets.

This phrase was modified accordingly: "Delaying the emergence of resistance will require the identification of new anti-plasmodial modes-of-action, ideally with low propensity for resistance."

4) Line 53. As noted above, “in vitro evolution of resistance” should be explained with first usage in the text.

This phrase was modified as follows: “While targets are not necessarily known at this stage in the process, approaches such as the *in vitro* evolution of resistant parasites using low drug pressure ranging from sub-lethal to $3\times IC_{90}$, followed by whole-genome sequencing to identify newly-generated mutations, can be used to deconvolute targets as well as compound mode of action [5,6]”

5) Figure 1. In the chemical structures shown, the orientation of compound 31 is flipped compared to that of the other structures; this won’t bother synthetic chemists, but makes comparison a bit more difficult for non-chemists. Showing the structures in the same orientation is preferred.

The non-chemists among us agree! The relevant structures have been modified accordingly.

6) Line 147. “Only one gene, PF3D7_1359900, was common to all clones” is obviously false; all clones shared thousands of genes. Rather, the authors meant to say that only one gene had unique mutations in all resistant clones.

That is indeed what we meant – modified accordingly.

Reviewer #2:

Part I - Summary

Please use this section to discuss strengths/weaknesses of study, novelty/significance, general execution and scholarship.

Reviewer #2: This manuscript by Rawat et al describes the discovery of potent antiplasmodial compounds based on quinoxaline-based schistosomal inhibitors. In fact, two of the analogues, 31 and 33, exhibit impressive single digit nM potency against both Dd2 and 3D7 as well as artemisinin partial resistant Cambodian strains. These compounds were shown to be irresistible even in the mutator strain. While the compound 22 which is slightly less potent generated modest resistance with point mutations in a non-essential gene PfQRP1. Although loss-of-function mutations suggest a mechanism of action via PfQRP1, compound 22 also yielded CNVs PfATP2 implying its role. In summary, the work presented describes the identification of potent antiplasmodial compounds with high barrier to resistance exhibiting complex yet novel mechanism of action that is distinct from that of current antimalarials.

Strengths: The research presented here is an important contribution to the malaria drug discovery efforts. Focus of the work is clearly to define the mechanism of action of these promising antiplasmodials.
Weakness: In vivo efficacy of these promising compounds would have made the story complete.

Part II – Major Issues: Key Experiments Required for Acceptance

Please use this section to detail the key new experiments or modifications of existing experiments that should be absolutely required to validate study conclusions.

Reviewer #2: None.

Part III – Minor Issues: Editorial and Data Presentation Modifications

Please use this section for editorial suggestions as well as relatively minor modifications of existing data that would enhance clarity.

Reviewer #2: The compounds should be referred to as antiplasmodials (including the title) as no antimalarial property has been established in the rodent model.

Many thanks for your consideration of our article.
We have modified all relevant usages of antimalarial throughout.

Reviewer #3:

Part I - Summary

Please use this section to discuss strengths/weaknesses of study, novelty/significance, general execution and scholarship.

Reviewer #3: The study identifies a novel chemotype - quinoxaline as having anti-malarial activity. The manuscript describes the anti-Plasmodial activity, a mechanism of resistance and a putative mechanism of action of quinoxaline-containing compounds. Quinoxaline-containing compounds are contained in the MMV Malaria Box, suggesting this chemotype has potential for anti-malaria drug development. The quinoxaline-containing compounds interrogated in this study were originally identified for their anti-Schistosomal activity. Hence this study is an interesting example of pathogen hopping. The authors tested their collection of quinoxaline-containing anti-Schisto compounds and found that 3 are active against the P. falciparum lab strain, 3D7. One of the 3 has limited activity against drug-resistant strains, the second maintains moderate activity and the third is highly active. In an attempt to gain insight into potential modes of action and mechanisms of resistance, the authors conducted evolved resistance studies using the 3 compounds. Resistance could not be selected against the most potent compound. Using parasites resistant to the other 2 compounds, the authors propose PfQRP1 as a mediator of resistance and PFATP2 as the direct target of the compounds. However there is no activity assays with PFATP2 to support its role as a target of this novel chemotype.

Thank you for the constructive points, which are addressed below.

Part II – Major Issues: Key Experiments Required for Acceptance

Please use this section to detail the key new experiments or modifications of existing experiments that should be absolutely required to validate study conclusions.

Reviewer #3: The manuscript is clearly written and data presentation is excellent.

My major concern centers on the identification of PfATP2 as the likely target. While additional experimentation may show this to be true, the current case is quite weak. It relies of the putative essentiality of PfATP2, which is itself is less than conclusive. Negative results in transfection experiments can be due to technical issues rather than essential function of the target gene. Indeed the targeting vector for P. berghei homolog of PF3D7_1219600 is annotated as having short recombination arms that can easily result in a lower rate of recombination at the locus. Similarly, piggybac transposon mutagenesis in P. falciparum has many false positives. The authors can conduct robust genetic targeting of PFATP2 (TetR=DOZI, DiCre) to determine whether or not it is essential. In addition to demonstrating essentiality of PFATP2, functional assays are needed to demonstrate that it is the target of the compounds, at least in vitro.

We agree that screening approaches such as piggybac or the PlasmoGEM *P. berghei* pooled insertion are not definitive for essentiality. We also cite the Kenthirapalan et al., (2016, Nat Comm, PMID: 26796412) study that performed individual knockout transfections in *P. berghei* – in their study homology arms for all their knockout vectors targeting 35 transporters were in the range of 400-650 bp (compared with 746/2084bp for the PlasmoGEM vector), of which the majority were successful in generating KOs.

We agree that a more compelling argument would be the generation of a conditional knockdown line to show both impact on growth and effect on compound sensitivity. We did not pursue the generation of this *pfatp2* conditional line here as we were aware of parallel work in the lab of Dr. Adele Lehane (ANU), who was performing these studies and as such we did not want to duplicate effort or compete with a close colleague. Their study, which will be submitted to bioRxiv shortly, shows that knockdown of *pfatp2* mRNA using the glmS ribozyme system results in a corresponding growth defect. Furthermore, *pfatp2* knockdown parasites are sensitised to MMV665794 and MMV007224, two of the compounds we describe here as analogs of compounds **22**, **31** and **33**. We have cited their study now in the Discussion (page 12, paragraph 1 of the tracked-changes version).

As additional evidence of essentiality, we have now also described the study by Patel et al., (mBio, 2022, PMID: 35862778) that attempted to generate conditional knockouts of the three *cdc50* genes (*cdc50a-c*) in *P. falciparum*. Lipid flippases typically require a CDC50 subunit for activity, and using the DiCre-loxP approach Patel et al showed that of the three *cdc50* genes, only *cdc50c* was essential. Furthermore, using immunoprecipitation they showed CDC50C forms a complex with PfATP2, consistent with CDC50C being the flippase partner and this activity being essential for the parasite (page 12, paragraph 1).

Another concern is the extent to which PfQRP1 is responsible for resistance to compounds 22, 31 and 33. Compound 33 was not tested against gene-edited parasites. 22 and 31 have quite different profiles in the Cambodian strains suggesting multiple mechanisms of resistance, some shared and others unique to 31. What is the status of PfQRP2 and PfATP2 in the Cambodian strains? If PfQRP2 mutations are absent in these strains then the significance of PfQRP2 as a resistance-mediator is reduced.

We have now performed assays with compound **33** against a variety of parasite lines, specifically the compound **22** drug selected parasites (new Fig.S5), the PfQRP1 edited parasites (PfQRP1-stop and PfQRP1-D1829A; Fig.5c) and the *pfatp2* CNV parasites (Fig.4c). Curiously, the PfQRP1 mutant lines do not show a shift with compound **33**, however the *pfatp2* CNV parasites do show a modest shift, significant only for the R2 line (see Fig.4c). This suggest compound **33** may be able to partially elude the resistance mechanism of PfQRP1, but nonetheless may act via the PfATP2 flippase, consistent with this being the target. We further compared cross-resistance of the compound **22**-selected clones with compound **33** and its structural isomer (compound **32**), as well as compound **30** (isomer of compound **31**) and compound **35** (new Fig. S5). All but compound **33** show a modest but significant shift against one of the compound **22**-evolved clones. In addition to inserting this new data, we briefly address this in the Discussion (page 11, paragraph 1).

To see if geographically distinct isolates have different responses to the compounds, we have also expanded the panel of wild type parasite strains tested against compounds **22**, **31** and **33** (see Fig.1c). In addition to Dd2 and the two Cambodian isolates, we have now included parasites from Uganda, Tanzania and Ghana. However none of the strains, including the two Cambodian lines, show significant differences in IC_{50} for each of the compounds compared with Dd2. Thus we do not think that any sequence differences between the different strains would yield functional information about how PfQRP1 or PfATP2 act.

Notwithstanding the ability of compound **33** to bypass PfQRP1 resistance, overall we believe that the data collectively indicate that PfQRP1 is a resistance mediator for quinoxaline-based

compounds more broadly. Resistance selections with compound **22** yielded two distinct mutations in PfQRP1, CRISPR generation of a truncation or putative catalytic triad mutation also yielded resistance to compounds **22** and **31** as well as MMV665794 and MMV007224, and previous resistance selections using these related compounds (MMV665794 and MMV007224) yielded additional mutations in PfQRP1 that conveyed resistance to a further 10 quinoxaline-based analogs (Kumpornsin et al., 2022, Nat Comm, PMID 37244916).

Part III – Minor Issues: Editorial and Data Presentation Modifications
Please use this section for editorial suggestions as well as relatively minor modifications of existing data that would enhance clarity.

Reviewer #3: (No Response)
