# Supplementary material for: Quinoxaline-based anti-schistosomal compounds have potent anti-plasmodial activity
Source: PLoS Pathog. 2025 Feb 3;21(2):e1012216. doi: 10.1371/journal.ppat.1012216 (PMC11809919; doi:10.1371/journal.ppat.1012216)
Supplement: Attachment — Submitted filename: Response_to_reviewers.docx [file ppat.1012216.s017.docx]

**Response to editorial and reviewer questions**

**Additional Editor Comments:**

The MS has been reviewed again following revision and resubmission with the author’s response to reviewers. A concern of original reviewer 1 was that the mechanisms of resistance identified were incomplete: Studies of likelihood of resistance and mechanisms of resistance are of important for compounds likely to be advanced as antimalarial drug candidates. The reviewer had concerns that that the mechanisms of resistance of the studied quinoxalines were not sufficient.
A similar concern was expressed by original reviewer 3 and expressed again following revision 1. The concerns were whether PfATP2 is essential and the actual target of these compounds. Also, there were concerns that there was no biochemical assay, and the genetic data cited was weak because the essential nature of CDPK1 not being confirmed.
I have looked again to search for recent bioRxiv for Adele Lehane and PfATP2 but not found it to support the evidence that PfATP2 is essential. Thus, it is my opinion that the manuscript as it stands, without clearer evidence of the mechanisms of resistance, does not fully meet PLoS Pathogen’s publication criteria as it stands.
1) The authors should genetically target PFATP2 (e.g. TetR=DOZI, DiCre) to determine if it is essential as has been suggested by reviewer 3 and alluded to by the original referee 1.
2) The authors should also include a relevant biochemical assay showing that it is the target of the compounds.

We agree these are important points. As noted in our previous submission, we were aware of work from the Lehane lab (ANU) that addresses both of these questions, which at the time of our resubmission was nearly complete and thus we did not want to duplicate efforts. Their excellent study has now been submitted to bioxRiv (<https://www.biorxiv.org/content/10.1101/2024.12.23.630022v1>), which we have also uploaded as an additional review item.

In brief, they show that:

i) conditional knockdown of PfATP2 using the glmS ribozyme results in a growth arrest that is correlated with a reduction in *pfatp2* mRNA level (Figure 1).

ii) knockdown of PfATP2 sensitizes parasites to two of the quinoxaline compounds described in our study (MMV007224 and MMV665794), whereas overexpression of PfATP2 confers resistance (Figure 2). These findings are consistent with PfATP2 being the target of these compounds.

iii) uptake of a fluorescent lipid substrate (NBD-PS) into saponin-lysed parasites is reduced in the PfATP2 knockdown (Figure 5A), consistent with a function for PfATP2 in lipid uptake.

iv) Both MMV007224 and MMV665794 compounds inhibit internalization of the NBD-PS lipid in the saponin-lysed parasite assay (Figure 6A,B).

Collectively their data are fully consistent with the model we suggest, which is that PfATP2 is the target of these quinoxaline-based compounds and that they inhibit the uptake of lipid species. We have updated our Discussion (lines 371-377) to cite this study and more fully discuss their findings.

**Reviewers' Comments:**

Reviewer's Responses to Questions

**Part I - Summary**
Please use this section to discuss strengths/weaknesses of study, novelty/significance, general execution and scholarship.

Reviewer #2: The manuscript reports antiplasmodial properties of quinoxalines and alludes to its mechanism of action. The authors have responded well to comments from the previous submission.

Thank you for your consideration.

Reviewer #3: (No Response)

Reviewer #4: This manuscript reports on the activities of anti-schistosomal quinoxaline molecules repurposed to target malaria. This is the first time I have reviewed this manuscript and note that it has been previously reviewed and revised. The authors determine the activity of “compound 22” in standard asexual growth assays and then demonstrate increased potency of analogues “31” and “33”. They demonstrate that all have similar activites against a range of different parasite isolates from different geographical (and drug sensistivity) backgrounds. The research team behind this work are renowned for their previous groundbreaking studies into determining antimalarial molecule mode of action/mechanism of resistance and they employ their well-validated pipeline to probe the quinoxalines. Through drug pressure to compound 22, modest (but statistically significant) resistance was generated and through genome sequencing, PfQRP1 mutations were implicated. Two lines possessing mutations in this gene were tested and they recapitulated the resistance phenotype. However, as PfQRP1 is non-essential, the authors conclude that this is likely a mechanism of resistance rather than the molecular target. Cleverly, the authors also generated a PfQRP1 mutant with an inactive catalytic triad that was substantially more resistant to compound 22 and 31, but not 33. Having detected copy number variants for the flippase gene pfatp2 the authors also note that this gene also contributes to resistance.

Reviewing this manuscript at this late stage, previous reviewers have highlighted suggested improvements that the authors have met. This study is thorough and proposes a new class of molecules that are dual active against two significant parasites.

Many thanks for the positive feedback.

**Part II – Major Issues: Key Experiments Required for Acceptance**
Please use this section to detail the key new experiments or modifications of existing experiments that should be absolutely required to validate study conclusions.

Generally, there should be no more than 3 such required experiments or major modifications for a "Major Revision" recommendation. If more than 3 experiments are necessary to validate the study conclusions, then you are encouraged to recommend "Reject".

Reviewer #2: (No Response)

Reviewer #3: The authors did not address a major concern about pfatp2 being the target of compounds. There is no biochemical assay or genetic validation. Failure to obtain a KO is not evidence of essentiality. They refer to work by Dr Lehane in biorxiv but I was unable to find the preprint.

We are pleased to note that the complementary study by the Lehane lab is now on bioRxiv (<https://www.biorxiv.org/content/10.1101/2024.12.23.630022v1>), and we have also uploaded this as an additional review item.

Please see our more detailed reply to the Editor above describing how their findings address these key points, and how we have incorporated these into our revised Discussion.

Reviewer #4: No major revisions required.

**Part III – Minor Issues: Editorial and Data Presentation Modifications**
Please use this section for editorial suggestions as well as relatively minor modifications of existing data that would enhance clarity.

Reviewer #2: (No Response)

Reviewer #3: (No Response)

Reviewer #4: The manuscript is thorough and research is well conducted. Noting the comments of previous reviewers, it is my opinion that the authors have addressed previously raised comments and I do not have any major concerns myself. There is always more characterization that could be performed – such as in vivo efficacy in a rodent model (or humanized mice) but I believe that this would be out of the scope of the manuscript and could form the basis of future work if this compound was progressed with a view to further development. From a cell biology perspective, given that other recently reported antimalarials target lipid transfer (e.g PfSTART), it would be interesting to study the effects of the quinoxalines on cellular morphology (i.e. membrane transfer, formation of the amoeboid form etc). However these also could form another manuscript.

Thank you for the suggestions. We agree that perturbation of lipid dynamics presents an interesting new area for antimalarial development, with many interesting starting points for further investigation. We have added this point to the discussion (lines 388-391) and referenced the PfSTART study.
